# Characterisation of buried blast loading

**DOI:** 10.1098/rspa.2019.0791

**Published:** 2020-04-29

**Authors:** Sam Clarke, Sam Rigby, Steve Fay, Andrew Barr, Andy Tyas, Matt Gant, Ian Elgy

**Affiliations:** 1 Department of Civil & Structural Engineering, University of Sheffield, Mappin Street, Sheffield S1 3JD, UK; 2 Blastech Ltd., The Innovation Centre, 217 Portobello, Sheffield S1 4DP, UK; 3 Defence Science and Technology Laboratory (Dstl), Porton Down, Salisbury, Wiltshire SP4 0JQ, UK

**Keywords:** buried explosive, confinement, Hopkinson pressure bar, pressure distribution

## Abstract

While it is well known that detonation of shallow-buried high explosive charges generally results in above-surface loading which is greatly amplified compared with the same detonation in air, uncertainty persists as to the mechanisms leading to this effect. The work presented in this paper is a systematic investigation into the mechanisms of load transfer in buried blast events. This paper details the results from a parametric study into the mechanisms and magnitudes of load transfer following a shallow-buried explosion, where spatial and temporal load distributions are directly measured on a rigid surface using an array of Hopkinson pressure bars. In particular, the investigation has looked at the influence of both geometrical confinement and geotechnical conditions on the loading. The parametric study was separated into four main threads: the influence of physical confinement; gravimetric moisture content; stand-off distance and depth of burial; and soil material/particle size distribution. This study allows a direct observation of the contributions of each of these distinct parameters, and in particular the ability to discern how each parameter influences the temporal form and spatial distribution of the loading.

## 1. Introduction

Due to the use of shallow-buried explosive devices in modern conflict zones, there is an increased need for the scientific community to understand the mechanisms that influence the loading generated when detonation products and surrounding materials impinge on a target. After a buried explosive detonates, a high-pressure wave travels into the surrounding soil, compacting the material as it propagates. The amount of energy lost to compaction of the soil medium is dependent on the properties of the soil and dictates how much energy is available to impart work to the target [1]. Once this pressure wave reaches the surface, the soil begins to move upwards as the detonation products continue to expand and drive the surrounding soil at supersonic velocities. This soil confines the detonation products until the ejecta has thinned sufficiently to allow the gases to vent, or the confined gases impact a target situated above the soil surface. The combined soil throw and focusing of the detonation products generally results in a higher magnitude and more spatially localized imparted load on the target than if the explosive were detonated in free air.

The magnitude of the total impulse imparted to a target has been shown to be influenced by geotechnical conditions such as moisture content/saturation [2–12], air voids and bulk density [6–14], and particle size distribution (PSD) [7–12], and by physical parameters such as depth of burial (DOB) [2,3,7,12,15,16] and stand-off distance (SOD) [3,6,15,17–19]. However, while the effects of many geotechnical and physical parameters on *total* impulse have been quantified experimentally, to date there have been limited experimental studies on the *localized* development of load on a target [20–23], and none at larger scale. This can be attributed to two main factors: the lack of a robust experimental technique for recording accurate pressures and impulses from buried explosive events; and difficulties associated with carefully controlling the geotechnical test bed. Thus, while numerical-based studies, e.g. [24], have suggested mechanisms which dictate the form and magnitude of the load from a buried explosive, experimental studies of these mechanisms have, to date, been limited.

Recently, the authors have improved the procedure for preparation of the soil bed in buried explosive trials, enabling the geotechnical test beds to be prepared with variations in bulk density of less than 2% and variations in moisture content of ±0.05–0.1%. This methodology was initially developed for half-scale testing [25] and has been successfully adapted for quarter-scale testing [26]. In conjunction with the use of the discrete pressure measurement apparatus detailed in [27], this enables a detailed scientific study into the precise influence of differing levels of geometrical confinement, and increasing soil moisture content, to be conducted. This paper reports amalgamated results from 40 tests conducted over a 3-year period.

## 2. Experimental work

### (a) Apparatus

Experiments were conducted at the University of Sheffield Blast & Impact Laboratory in Buxton, Derbyshire, UK. The Characterisation of Blast Loading (CoBL) apparatus is described in detail in [27] and consists of an array of Hopkinson pressure bars (HPBs) [28] whose faces lie flush with the surface of an effectively rigid 100 mm thick, 1400 mm diameter steel target plate, mounted to a large, stiff, steel fibre and bar reinforced concrete frame. The 3.25 m long, 10 mm diameter EN24(T) steel HPBs were suspended from a small receiver frame placed on top of the concrete reaction frame to enable fine adjustments to be made to the heights of the HPBs. Semi-conductor strain gauges were mounted in pairs on the perimeter of each HPB, 250 mm from the loaded face, in a Wheatstone-bridge circuit to cancel out any bending stresses and only record axial stresses acting in the bars. Mounting the impacted face of each HPB flush with a reflecting surface means that the reflected pressure generated on that surface is applied to the bar, and also that the sensitive recording gauge station is protected from the aggressive loading environment. Recordings were triggered via a voltage drop in a break wire embedded in the explosive to synchronize the traces with the time of detonation.

In the standard arrangement, the loaded face of the target plate is situated 140 mm above^1^ and co-axial to a 500 mm diameter, 375 mm high steel container made from 30 mm rolled steel plate. The HPBs are arranged in four perpendicular arrays at a centre-to-centre spacing of 25 mm along each array, out to a distance of 100 mm from the plate centre, with one central bar common to all four arrays. This gives four data points at each radial distance of 25, 50, 75 and 100 mm, plus a singular value at the centre of the plate, for each test.

### (b) Scaled representation of the threat

The standard testing arrangement in this study is geometrically similar to a quarter-scale version of STANAG threat level M2b as given in the Allied Engineering Publication *Procedures for evaluating the protection level of logistic and light armoured vehicles (AEP-55)* [29], a testing addenda to the NATO standardization agreement, STANAG 4569 [30]. Several changes have been made from the conditions specified in STANAG M2b. Firstly, the 6 kg TNT charge specified for full-scale testing, formed into a 3:1 diameter:height cylinder, has been replaced with an equivalent full-scale charge of 5 kg PE4, with the same geometry, assuming a TNT equivalence of 1.2 [31], giving a 78 g charge at quarter scale. Secondly, the scaled DOB of 25 mm has been maintained, but with an additional 3 mm of soil overburden provided to account for the removal of the lid of the 3 mm thick PVC casing. This lid was removed to reduce signal noise caused by fragmentation which was observed in preliminary testing [32]. Finally, the baseline-scaled SOD from the soil surface to the target was set at 140 mm to prevent potential yielding of the HPBs (this was decreased to 105 mm in Series H, once the magnitude of the loading had been assessed). The geometry of the standard arrangement is shown in figure 2*a*, and is labelled ‘Series D’ in table 1.

**Table 1.** Summary of experimental test plan (note: for ‘air’ and ‘surface’ confinement tests, SOD refers to the distance from the top of the charge to the target, whereas for ‘partial’ and ‘full’ confinement, SOD refers to the distance from the top of the soil surface to the target, figure 2).

series	test nos.	fill material	moisture content (%)	bulk density (kg m^−3^)	confinement	DOB (mm)	SOD (mm)
A	1–2	air	—	1.225	air	—	168
B	3–4	LB	2.5	1640	surface	—	168
C	5–6	LB	2.5	1640	partial	28	140
D	7–11	LB	2.5	1640	full	28	140
E	12–14	LB	5.0	1670	full	28	140
F	15–17	LB	25*	1990	full	28	140
G	18–19	water	100	1000	full	28	140
H	20–24	LB	2.5	1640	full	28	105
I	25–29	LB	2.5	1640	full	28	175
J	30–33	LB	2.5	1640	full	53	140
K	34–37	Stanag	14*	2220	full	28	140
L	38–40	clay	56*	1750	full	28	140

Soils marked with * are fully saturated.

AEP-55 specifies that the soil container is filled with a well-graded sandy gravel. In this study, the standard testing arrangement uses 14/25 graded Leighton Buzzard (LB); a rounded to well-rounded quartz silica sand with a relatively uniform PSD (0.6–1.18 mm), although a sandy gravel is used for one particular series of tests to assess the influence of soil type. In the standard tests, the LB was specified to have a gravimetric moisture content, *w*, of 2.5% when compacted to a bulk density, *ρ*, of 1640 kg m^−3^, where *w* is defined for soils as the mass of water divided by the dry mass of solids. Although in a number of tests *w* was increased, the LB soil was compacted to a consistent *dry* density, *ρ*_*d*_, of 1600 kg m^−3^ throughout. The exact mass of fill material was added to the soil container (pre-compaction) to achieve the desired soil surface height, according to the soil preparation and compaction methodology detailed in [26]. LB has been shown to exhibit the lowest test-to-test variation when conducting tests with buried explosives, and is, therefore, best suited to scientific study [25].

In a number of tests, the fill material was replaced with one of three materials: water; a well-graded sandy gravel (0.2–20 mm particle size) termed ‘Stanag soil’ which falls within the basic parameters prescribed in STANAG 4569 [30]^2^; and a highly refined kaolin of ultra-fine particle size (0.5% >10 μm by mass, 76–83% <2 μm) produced from deposits of Speswhite china clay in South West England [33]. These soils were prepared according to the methodologies for cohesionless and cohesive soils, respectively, as outlined in [34].

**Figure 1.**
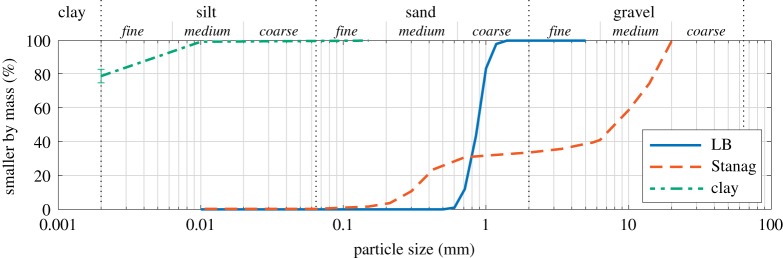
Particle size distributions of Leighton Buzzard sand (LB) and well-graded sandy gravel (Stanag) from sieve analysis, and kaolin clay from manufacturer’s specification [33]. (Online version in colour.)

Figure 1 shows the PSD of the three soils, where the LB and Stanag curves were determined from sieve analysis and the clay curve was taken from the manufacturer’s specification [33]. Here, ‘smaller by mass’ denotes the relative mass of soil passing through each sieve and therefore the percentage of soil particles by mass that are smaller than that particular particle size.

### (c) Test plan

The test plan can be described by four parametric studies, each of which contains a number of individual test series. In total, 40 tests were conducted. These are detailed below and are summarized in table 1.

#### (i) Influence of degree of confinement: Series A–D

**Figure 2.**
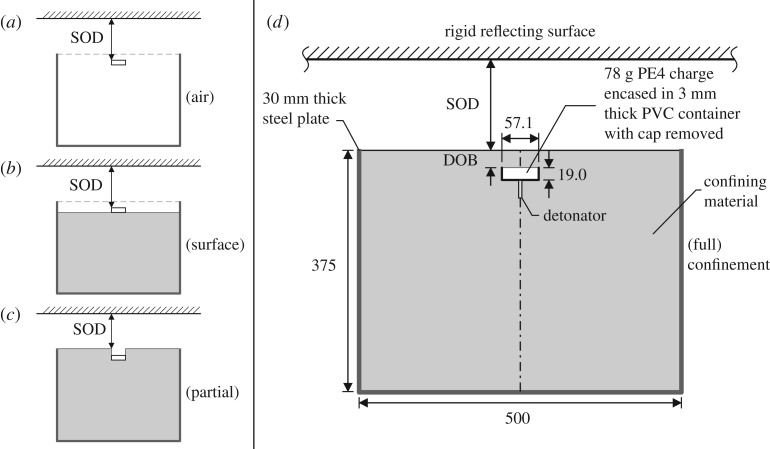
Definitions of different confinement levels and geometry of test arrangement (lengths in mm): (*a*) free air in Series A, (*b*) surface placement in Series B, (*c*) partial confinement in Series C and (*d*) full confinement in Series D–L.

A: Charge within an empty soil container,^3^ with 168 mm from the top of the charge to the underside of the target, as in figure 2*a*.
B: Charge sat directly on top of an LB (*w* = 2.5%) soil surface, with 168 mm clear distance from the top of the charge to the target. Soil surface 190 mm beneath the target (accounting for 3 mm PVC casing beneath the charge), as in figure 2*b*.
C: Charge situated as if it were buried in LB (*w* = 2.5%) to a depth of 28 mm (soil surface to top of charge), with material above the charge removed such that the sand only offers lateral confinement, as in figure 2*c*.
D: Charge buried in LB (*w* = 2.5%) to a depth of 28 mm (soil surface to top of charge). Soil surface 140 mm beneath the target, as in figure 2*d*. This represents the standard testing arrangement and is used to compare the influence of degree of confinement and geotechnical conditions.

#### (ii) Influence of moisture content: Series D–F

D: Baseline condition (*w* = 2.5%).
E: As D, with *w* = 5.0% (*ρ* = 1670 kg m^−3^, *ρ*_*d*_ = 1600 kg m^−3^).
F: As D, with *w* = 25% (*ρ* = 1990 kg m^−3^, *ρ*_*d*_ = 1600 kg m^−3^).

#### (iii) Influence of DOB and SOD: Series D, H–J

D: Baseline condition (SOD = 140 mm).
H: As D, with SOD reduced to 105 mm.
I: As D, with SOD increased to 175 mm.
J: As D, with DOB increased to 53 mm.

#### (iv) Influence of confining material: Series F, G, K, L

F: Baseline condition (LB, *w* = 25%, *ρ* = 1990 kg m^−3^).
G: As F, with the container filled with water at room temperature. The charge was situated on a small timber prop within the container to accurately control the submerged depth.
K: As F, with the container filled with Stanag soil (*w* = 14%, *ρ* = 2200 kg m^−3^).
L: As F, with the container filled with kaolin clay (*w* = 56%, *ρ* = 1750 kg m^−3^ [35]).

### (d) Interpreting pressure-time data

**Figure 3.**
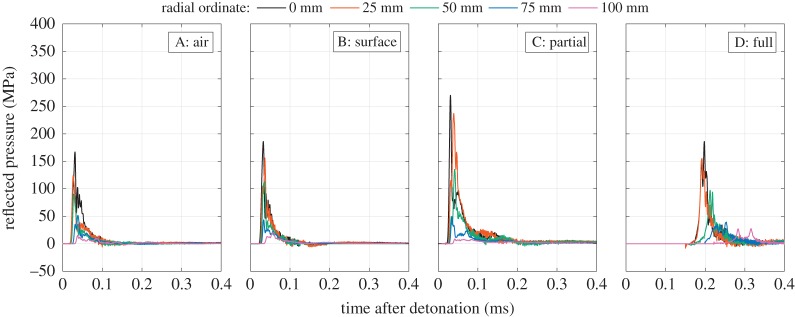
Pressure-time histories: Series A–D: influence of confinement. (Online version in colour.)

Figure 3 shows example pressure-time histories for Series A–D. Here, a single array from 0 to 100 mm from the target centre is shown for a single test in each series. The time datum has been shifted by −0.05 ms to account for the time taken for the pressure pulse to travel from the loaded face of the HPB to the strain gauge location. The pre-trigger voltage has been subtracted from each signal, hence the pressure terms described hereafter relate to overpressures (i.e. pressure above ambient).

Some high-frequency oscillations can be seen in the traces as a result of Pochhammer–Chree dispersion. When designing the CoBL apparatus, the effects of Pochhammer–Chree dispersion [36–38] were minimized by selecting a small diameter bar relative to the physical scale of the apparatus, and fixing strain gauges as close to the loaded face as practically possible. Hence, the effects are minimal for this test arrangement, and peak-specific impulse is unaffected. As such, dispersion correction has not been performed. Other than the time-bases being shifted as described above, the traces are otherwise presented ‘as-recorded’ and have not been smoothed or filtered.

A full set of compiled reference results for each series are contained within the electronic supplementary material.

## 3. Results and discussion

### (a) The influence of degree of confinement: Series A–D

The first part of the parametric study is focused on quantifying the effects of lateral confinement offered by a surrounding soil mass on the localized pressure acting on a rigid target after detonation of an explosive charge.

Despite varying levels of confinement and back-tamping being offered by the soil, the pressure histories for Series A (air), B (surface) and C (partial) in figure 3 all appear to resemble the well-known ‘Friedlander’ exponential waveform typically associated with free-air blast events [39] and seen in experimental recordings of near-field blast pressures from spherical charges [40,41]. The blast pressures exhibit a rapid rise to a single, well-defined peak, followed by an exponential decay afterwards. The waveforms in Series C are generally higher in magnitude and longer in duration than those in Series A and B, owing to the lateral confinement offered by the soil adjacent to and above the plane of the charge.

Conversely, while the pressure traces in Series D are similar in magnitude to those in Series A and B, the arrival time is delayed by around an order of magnitude as the inertial confinement of the soil overburden serves to slow vertical expansion of the detonation products. In Series D, the loading is similar to a Friedlander exponential which has been spread, both in space and time, when compared with the free-air or partially confined charges in Series A–C. This difference is perhaps clearest when comparing the pressure trace from the 100 mm bar in Series D with the traces from the 100 mm bars in Series A–C.

**Figure 4.**
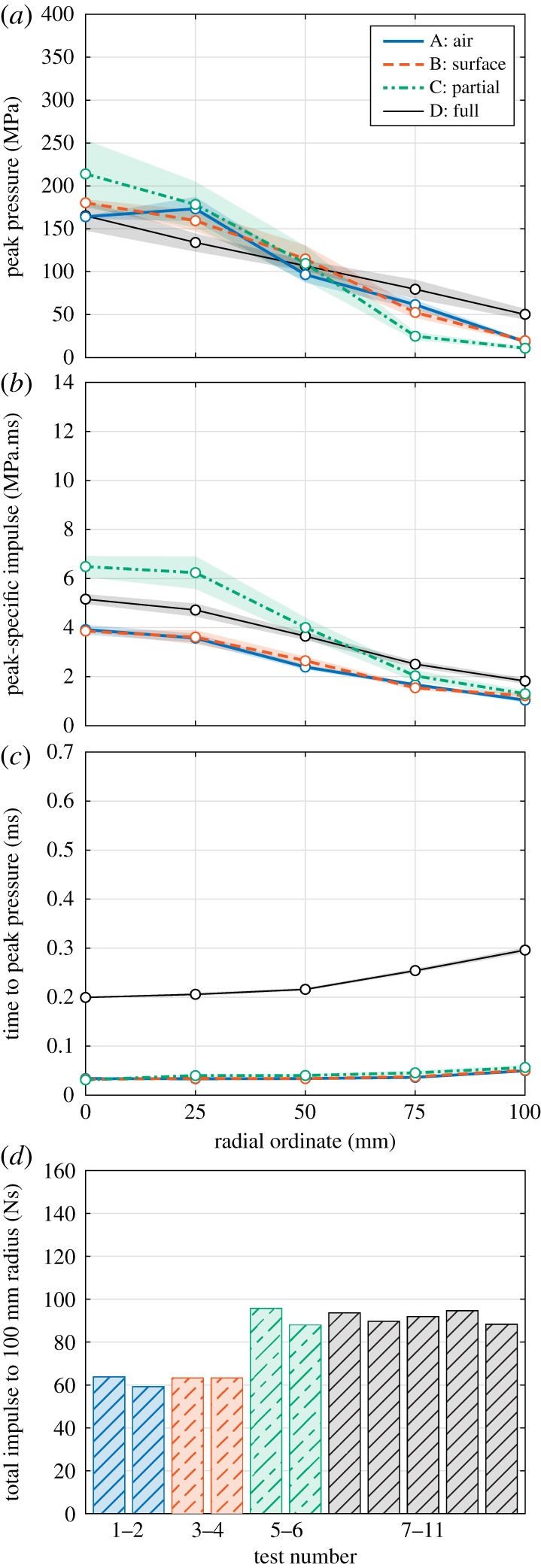
Influence of confinement (Series A–D): radial variation of (*a*) peak pressure, (*b*) peak-specific impulse and (*c*) time to peak pressure, and (*d*) area-integrated impulse. Shading is standard error of the mean. (Online version in colour.)

**Figure 5.**
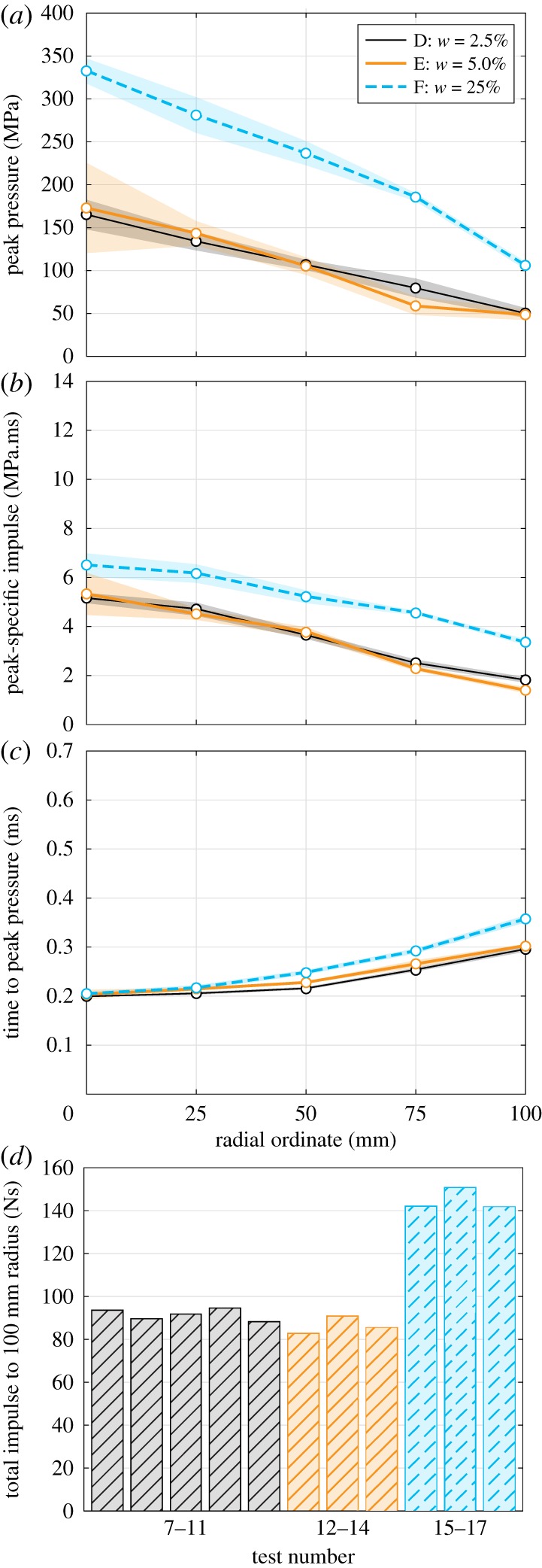
Influence of moisture content (Series D–F): radial variation of (*a*) peak pressure, (*b*) peak-specific impulse and (*c*) time to peak pressure, and (*d*) area-integrated impulse. Shading is standard error of the mean. (Online version in colour.)

To enable a meaningful comparison between each series, rather than comparing sample pressure traces, figure 4 shows distributions of peak pressure (a), peak-specific impulse (b), and time to peak pressure (c)^4^ plotted against radial ordinate for each Series A–D. Here, specific impulse is the integral of the pressure history. The *y*-values in these subplots are given as the average of all bars at a particular distance from the plate centre for all tests within the series (data from one 0 mm bar and four 25, 50, 75 and 100 mm bars per test). The shaded area in each subplot denotes the standard error of the mean. Also shown is the total area-integrated impulse over the 100 mm radius instrumented region (d), taken as the integral of the peak-specific impulse curve assuming a linear distribution between each radial ordinate. Figure 4 is plotted alongside figure 5 to allow a direct comparison between the loading parameters in each parametric study.

The distribution of peak pressure across the central 100 mm radius of the target plate is more uniform when the charge is buried in soil (Series D, figure 4*a*). That is, the reflected pressure in Series D decays from a mean value of 165 MPa at the centre to 50 MPa at 100 mm, compared with a decay from 214 MPa at the centre to 11 MPa at 100 mm for Series C, and from 164–180 MPa to 18–20 MPa for Series A and B.

Interestingly, the specific impulse distribution from Series D appears to follow those of Series A and B, albeit with an additional ‘baseline’-specific impulse of approximately 1 MPa.ms, which gradually decreases with distance from the plate centre (figure 4*b*). This is consistent with previous observations that approximately 25% of the specific impulse recorded at the central bar location from explosives buried in dry LB was due to late-time particle barrage [26]. The specific impulse distribution in Series C is considerably less uniform than in Series A, B and D. This highlights the influence of the ‘focused path’ offered to the detonation products, and hence preferential venting towards the centre of the target, caused by removal of the soil above the charge.

There is a clear delay in arrival time between Series A–C and Series D (figure 4*c*), owing to the inertial resistance offered by the sand above the charge. The area-integrated impulses for Series C and D are similar (figure 4*d*), despite the distributions of specific impulse being considerably different. In this instance, it is clear that Series C-type loading would result in greater structural displacement than Series D-type loading [42,43], however, this would not be discernible if measuring total impulse only.

### (b) The influence of moisture content: Series D–F

The second part of the parametric study is focused on quantifying the influence of moisture content on the output of a buried explosive. While previous experimental studies have shown that total impulse increases with moisture content (e.g. [2,10,11,13]), the underlying mechanisms driving this increase and the effect on localized loading and loading distribution have yet to be studied.

**Figure 6.**
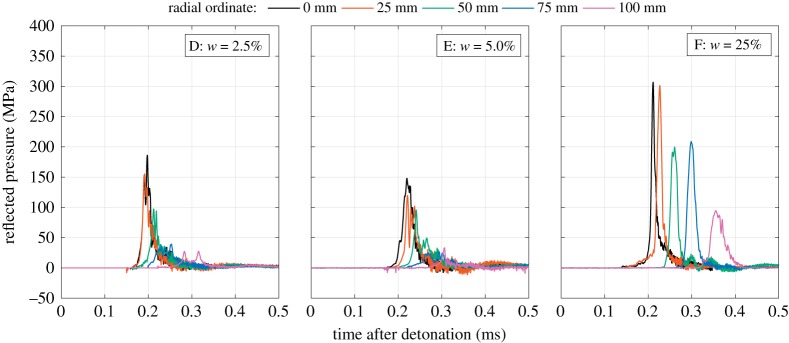
Pressure-time histories: Series D–F: influence of moisture content. (Online version in colour.)

When comparing the waveforms from Series D and E (both low-moisture content soils) with Series F (saturated soil) in figure 6, two key differences become apparent. Firstly, the loading from an explosive buried in saturated soil is much higher in magnitude than the loading from an explosive buried in low-moisture content soil at the same dry density. Secondly, there appear to be two distinct loading mechanisms for low-moisture content and saturated soils, respectively.

Generally, the pressure in the low-moisture content soils (Series D and E) exhibits a sharp rise to peak value followed by a more gradual decay to ambient pressure. As the pressure wave propagates along the target surface and reaches each radial bar location, the pressure is also seen to rapidly rise to peak pressure and subsequently equalize with the instantaneous pressure at the inner bar locations. High-speed video has shown that, in tests using low-moisture content soils, the soil bubble containing the detonation products ruptures prior to the soil reaching the target [26], which allows the high-pressure gases to vent, interact with, and spread across the target surface. Thus, the loading for low-moisture content soils largely resembles a delayed air shock with added short duration fluctuations due to the soil throw. At any instant in time, the loading is relatively uniform over the loaded area.

Conversely, the loading arising from the explosives buried in saturated soil (Series F) exhibits a rise to peak pressure, followed by a near symmetrical decay back to ambient pressure. This is consistent with the loading generated in the early stages of the impact of a high-velocity fluid drop with a solid surface [44]: as the radius of the contact surface between the droplet and the surface expands, the highest pressures are generated just inside the contact region. With increasing radial spreading of the fluid annulus, the angle between the fluid drop (or soil bubble) and the wall increases and the contact edge velocity decreases, reducing the magnitude of peak pressure. This behaviour is consistent with the pressure traces observed in the Series F tests and suggests that the soil bubble containing the detonation products is still intact by the time it reaches the target. At any instant in time, the blast pressure is highly non-uniform.

Figure 5 shows distributions of peak pressure, peak-specific impulse and time to peak pressure plotted against radial ordinate, and total area-integrated impulse over the 100 mm radius instrumented region for Series D–F.

While Series D and E are broadly similar, the peak pressure in Series F appears to decay more rapidly with increasing distance (figure 5*a*), which again is consistent with the behaviour of a pressurized fluid droplet impact. The specific impulse at the central bar is 25% larger in Series F than Series D and E, and is approximately 100% larger at the 100 mm bar location (figure 5*b*). This suggests that the approximate factor of two increase in total impulse (seen in the previous testing [2,10,11,13] and largely consistent with the area-integrated impulses in figure 5*d*) is as a result of a different loading mechanism, in addition to the added mass and confinement offered by a saturated soil. There appears to be a slight delay in the arrival time of the loading at the 100 mm bar location in Series F when compared with Series D and E (figure 5*c*), which suggests that the lateral flow of blast pressures across the target is slower when the soil is saturated.

### (c) The influence of DOB and SOD: Series D, H–J

The penultimate aspect of the parametric study is focused on quantifying the influence of physical parameters, e.g. DOB and SOD.

**Figure 7.**
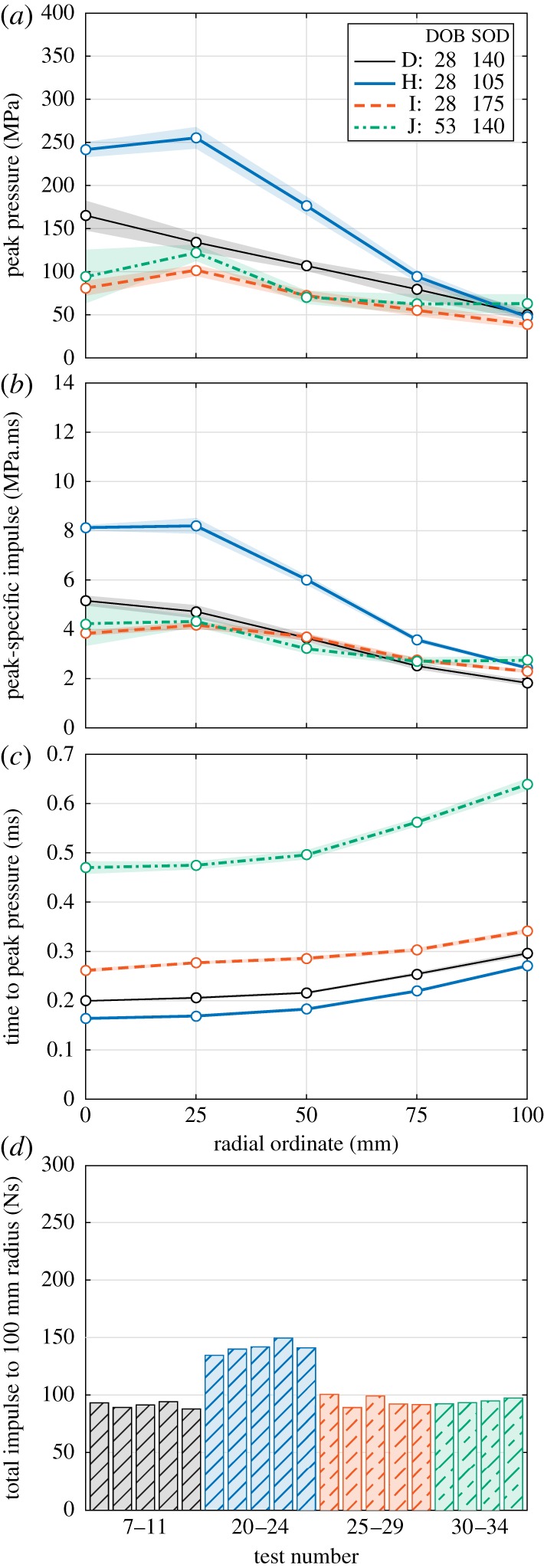
Influence of depth of burial (DOB) and stand-off distance (SOD) (Series D, H–J): radial variation of (*a*) peak pressure, (*b*) peak-specific impulse and (*c*) time to peak pressure, and (*d*) area-integrated impulse. Shading is standard error of the mean. (Online version in colour.)

The influence of SOD is clear when viewing the compiled parameters for Series H, D and I in sequence: as SOD is increased the loading becomes lower in magnitude (figure 7*a*,*b*) with an increased time to peak pressure (figure 7*c*).

The reduction in the distance from the soil surface to a target (Series H) results in a more centrally focused load: pressure and impulses for Series H are 50% larger than the Series D values at the central bar, but are comparable with the Series D values at the 100 mm bar location (figure 7*a*,*b*). This focused loading distribution results in a total area-integrated impulse which is, again, 50% larger than the Series D values (figure 7*d*), for only a 25% reduction in SOD and a 21% reduction in total distance from the charge to the target (table 1).

Increasing either the SOD (Series I) or the DOB (Series J) results in a more spatially uniform loading distribution (relative to Series D). Both Series I and J have similar pressure and impulse distributions, with smaller values at the central bar and larger values at the 100 mm bar relative to Series D. While the area-integrated impulses for Series D, I and J are all between 90 and 95 Ns, it is worth noting that over a larger area the Series I and J impulses would be larger owing to the flatter impulse distribution profile.

Arrival times are in order of increasing SOD and DOB, with DOB (Series J) seen to contribute more to a delayed arrival time compared with SOD (Series I) (figure 7*c*).

### (d) The influence of confining material: Series F, G, K and L

Finally, the influence of confining material was studied. Series F, G, K and L comprise tests with saturated LB, water, saturated Stanag soil, and kaolin clay (see §3b), in an attempt to understand how the bulk density and PSD of a soil influences the distribution of loading on a target.

**Figure 8.**
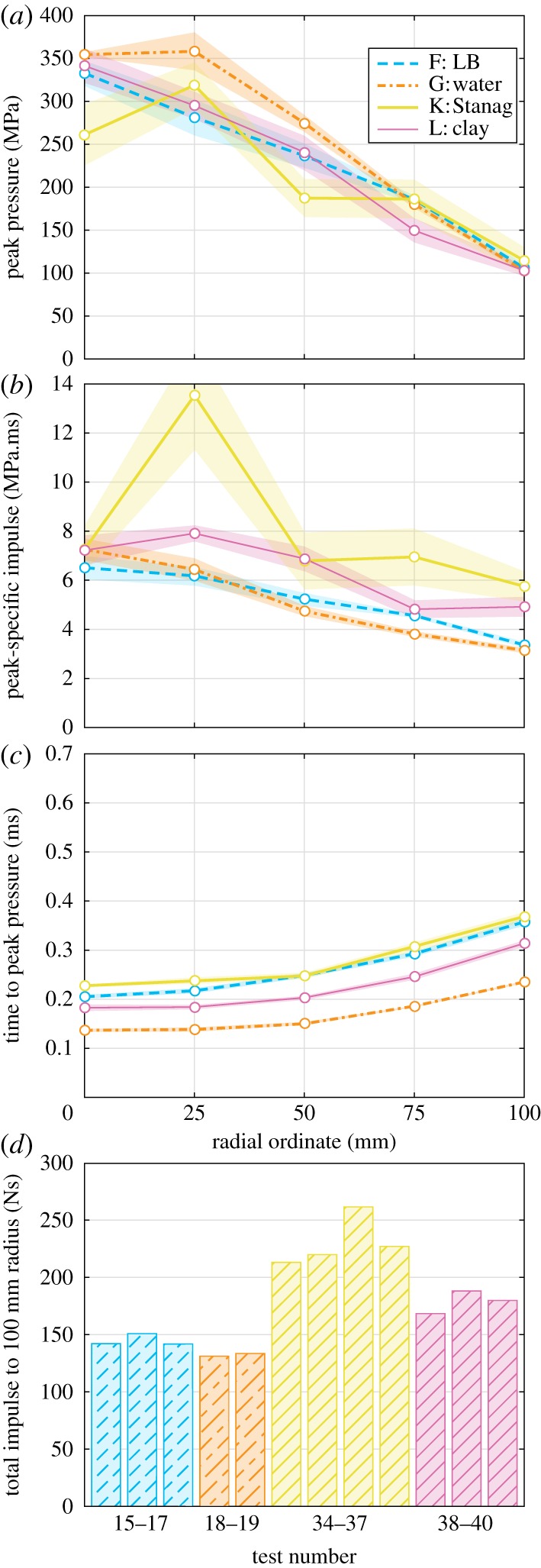
Influence of confining material (Series F, G, K and L): radial variation of (*a*) peak pressure, (*b*) peak-specific impulse and (*c*) time to peak pressure, and (*d*) area-integrated impulse. Shading is standard error of the mean. (Online version in colour.)

It can be observed that all the peak pressure distributions (figure 8*a*) appear similar in form, with the Series F and L pressure distributions (saturated LB and kaolin clay, respectively) matching particularly closely. That these distributions are similar to the Series G traces (water) suggests that the mechanism of loading for saturated soils is indeed due to a highly pressurized fluid impact, as opposed to the more variable, soil-entrained air shock loading seen in the low-moisture content soils. The saturated Stanag tests display some degree of experimental spread.

The specific impulse distributions (figure 8*b*) for Series F and G are again similar, with Series K and L exhibiting significant spread due to contributions from discrete particle strikes, which increases the specific impulse distributions above those from LB and water. This increased variability is hypothesized to be linked to the PSD of the soil, with well-graded soils (Stanag) exhibiting a greater variability, and uniform soils (LB) generating a more repeatable load [34]. This results in a significant increase in the area-integrated impulse (figure 8*d*); however, it is likely that this data is slightly skewed by assuming the contribution of the particle strikes is linearly averaged between bars at the same radial ordinate and should be taken as indicative only. Arrival times are in order of the bulk density of the confining material: water; clay; LB; Stanag. This suggests that the bulk density of the soil is dominant in determining the temporal characteristics of the loading (e.g. arrival time and loading duration).

## 4. Interpretation

### (a) Loading mechanism as a function of confinement

**Figure 9.**
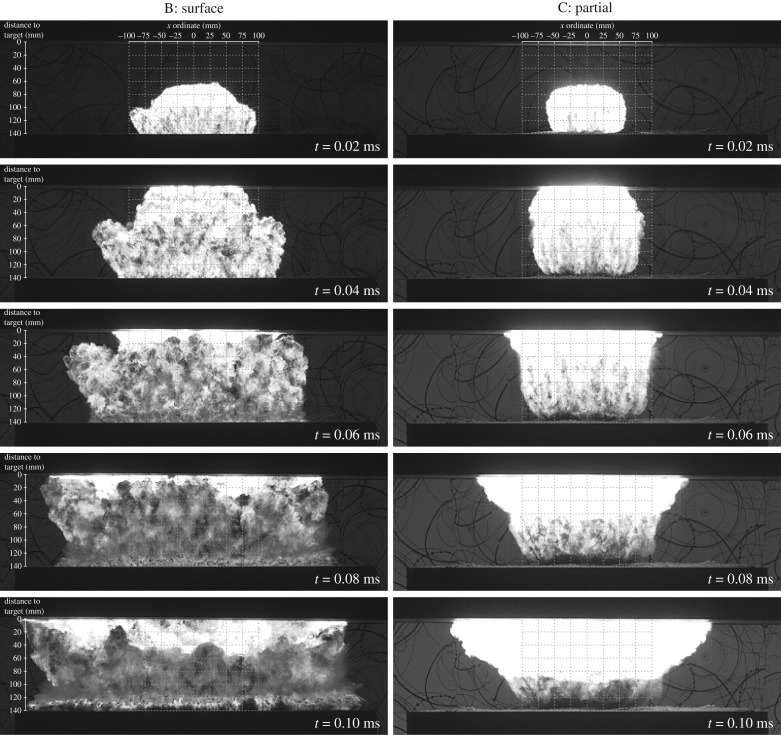
High-speed video stills from test 3 (Series B) and test 5 (Series C).

The most notable finding from these tests is that back-tamping the charge with dry sand (‘surface’ condition, Series B) produces no measurable difference in any of the loading parameters (figure 4) compared with the scenario where the charge is suspended in air (Series A). This is presumably due to the great acoustic impedance mismatch between the detonation products (before they have expanded) and the dry sand. The density of both materials are similar (1500–1600 kg m^−3^), but the wave speed is an order of magnitude or more higher in the detonation products than in the sand (approx. 10^3^ m s^−1^ compared with approx. 10^2^ m s^−1^). Consequently, the expansion of the detonation products downwards will tend to produce, in the initial stages at least, a crushing of the sand particles and compaction of the bulk material, rather than reflecting from the interface between the materials. This will lead to initial cratering of the soil surface, and as the soil becomes highly compacted, it may then be able to reflect more of the detonation products. High-speed video images from Series B tests suggests that this refection has a lateral as well as an axial component of momentum, possibly directing the reflected detonation products outside the measured area of the target (figure 9).

Conversely, the presence of dry sand both behind and around the side of the charge (‘partial’ confinement, Series C) results in a significant focusing effect of the detonation products towards the centre of the target, which in turn results in large increases in the blast load over the measured region. It is possible that the presence of the lateral confinement inhibits the lateral reflection of the detonation products and focuses them towards the central area of the target.

Full confinement of the charge in dry sand (Series D) produces similar magnitudes of overall impulse transmitted to the target as the partial confinement, but there are clear differences in the detailed loading parameters. In particular, the presence of sand above the charge results in the target being loaded by a combination of detonation products and sand. This additional inertia means that the impacting material expands at only approximately 20% of the rate seen in the less confined tests (figure 4*c*), but the impacting material is significantly more dense. Consequently, the resulting pressure-time signals are very slightly more rounded in form and less like the sharp-fronted Friedlander shock pulses than those where the detonation products impinge directly on the target (figure 3). The epicentral loads are lower than in the partial confinement case, but the magnitudes decay less rapidly with radial offset on the target.

### (b) Loading mechanism as a function of moisture content

The small increase in moisture content from 2.5% to 5% from Series D to E has little influence on the magnitudes and spatial distribution (figure 5*a*,*b*) or the temporal development (figure 3) of the loading pulses. At these levels of moisture content, the water is mostly adhering to the sand particles rather than filling interstitial gaps, and so, other than a very small change in bulk density, the geotechnical conditions are identical.

However, at full saturation (Series F), very different loading is observed. Quantitatively, the total impulse imparted to the target is around 50% higher than that recorded for drier sand (figure 5*d*), which is clearly linked to the higher pressure magnitudes seen in the saturated tests (figure 5*a*). Qualitatively, the temporal development of the loading pulses are entirely different from those seen in detonations in unconfined, partially confined or fully confined with dry soil conditions (figure 5).

While no comparable experimental study of the spatial distribution of loading currently exists in the literature, Grujicic & Pandurangan [24] observed two distinct loading mechanisms when performing numerical analyses of the loading generated from explosives buried in saturated and dry sand. They hypothesized that these different loading mechanisms are intrinsically linked to the formation, expansion, and rupture of the soil bubble confining the explosive material, and termed the two mechanisms ‘shock-type’ and ‘bubble-type’ loading. These are defined as follows:

— *Shock-type loading*. Dry sands have a lower cohesive strength and a thinner soil bubble compared with saturated soils. The bubble ruptures relatively early, allowing the detonation products to escape and impact the target. The loading, therefore, mainly comprises an air-shock load from the detonation products, with additional momentum transfer coming from later-time soil throw.
— *Bubble-type loading*. Saturated sand has a lower compressibility and higher cohesive strength, resulting in a larger volume of soil ejecta and delayed bubble rupture. This bubble confines the detonation products completely, and continues to do so for some time after it impacts the target and spreads laterally. Here, the loading is mainly comprised of momentum transfer from the soil/water mixture, driven by the more effectively confined detonation products.

**Figure 10.**
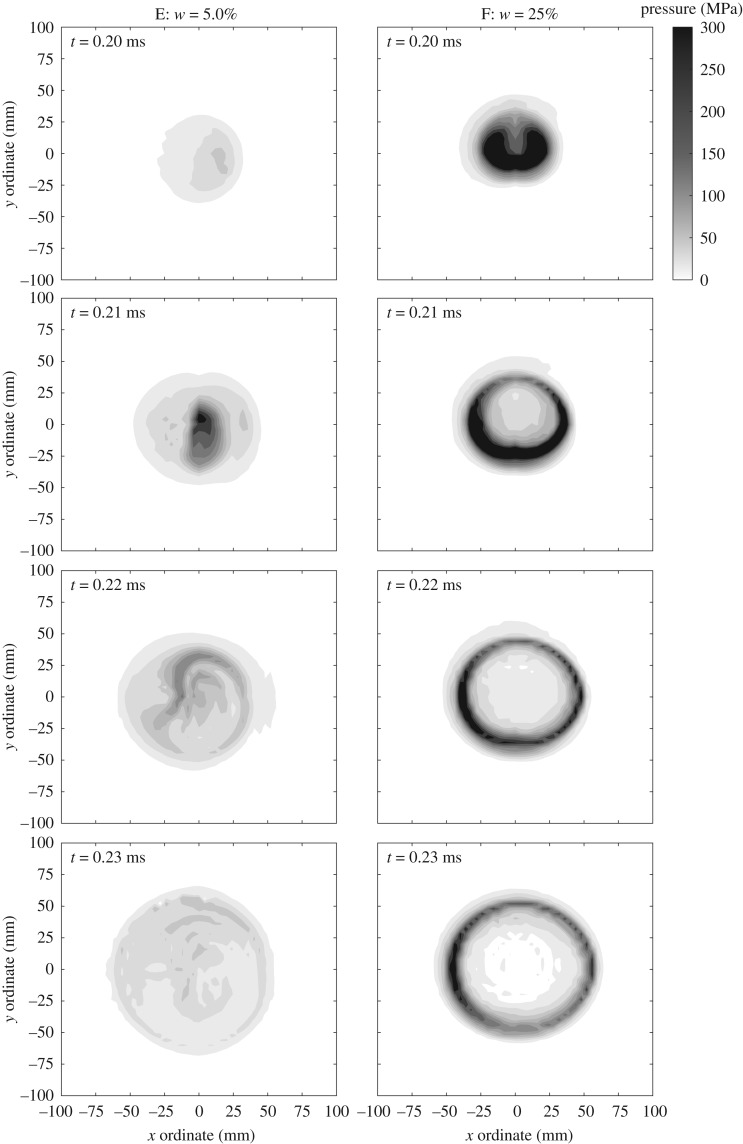
Interpolated pressure data for test 14 (Series E, *w* = 5.0%) and test 17 (Series F, *w* = 25%).

Figure 10 shows the pressure distribution acting over a central 200 mm square region of the plate. Here, the pressure distribution was calculated from interpolation of the experimental recordings from all 17 HPBs in Test 14 (Series E, *w* = 5.0%) and Test 17 (Series F, *w* = 25%), respectively, using the interpolation algorithm developed in [27].

The interpolated data reveal considerable fundamental differences in the spatial-temporal distribution of pressure loading from explosives buried in dry and saturated soils. In the dry/low-moisture content soils, the loading appears highly variable, albeit occupying a reasonably tight banding around some value of pressure which can be seen to act as a uniform background load. Conversely, the loading from the saturated soil appears as a thin shell of highly compressed material travelling outwards from the point of impact, with relatively little loading elsewhere on the target.

In addition, there are several aspects of the loading which appear consistent with the ‘shock-type’ and ‘bubble-type’ loading mechanisms described previously. Firstly, for the low-moisture content soils there appears to be a precursor shock front which reaches the HPBs some time before the main loading pulse. This is perhaps best seen in the pressure distribution for *w* = 5.0% where, for example, at *t* = 0.20 ms after detonation, a region of relatively low magnitude (less than 30 MPa), uniform loading extends from approximately 30 to 60 mm from the plate centre (figure 10). Secondly, the complexity of the combined soil/air loading in the low-moisture content soil is evident, with a large diameter region of relatively uniform pressure punctuated by sporadic spikes covering small areas of the target. Thirdly, there is a clear difference in the arrival time of the loading, with the low-moisture content soil not registering any significant load until some 10−20 μs after the arrival of the soil bubble in the saturated soil tests. This is presumably due to the early venting of the detonation products reducing the velocity of the soil/air mixture.

Bulk density, PSD, DOB and SOD are known to influence the shape and failure of the soil bubble. Series D–F provide definitive experimental evidence to support that these distinct mechanisms are observed in experimental testing.

## 5. Summary and conclusion

We have performed a detailed parametric study, comprising 40 tests at quarter scale, to study the mechanisms and magnitudes of loading resulting from detonation of an explosive buried in soils of varying composition, moisture content and geometrical confinement. Experiments were performed using the CoBL apparatus [27] housed at the University of Sheffield Blast & Impact Laboratory. The consistency of the test results, facilitated through very careful preparation of the soil beds, enables us to draw firm conclusions on the effect of the surrounding medium on the magnitudes and distributions of the loading on a target, and to discuss the nature of loading mechanisms. Key findings on the mechanisms underpinning the differences in loading are summarized below:

— The soil surrounding a charge serves to direct the detonation products upwards. The soil above the charge serves to spread the loading, in both space and time, and contributes an additional momentum transfer.
— The loading imparted to a target from an explosive buried in saturated soil is considerably higher than that from an explosive buried in a low-moisture content soil. This increase in magnitude—a factor of between 1.6 and 2.0 (based on current work and existing literature)—is considerably higher than the increase in bulk density (a factor of 1.2), which suggests that the loading is more complex than simply being a function of soil momentum alone.
— There is a fundamental difference between the loading mechanism for dry/near dry and for saturated soils. These are termed ‘shock-type’ and ‘bubble-type’ loading, and are characterized by: a sharp rise to peak pressure followed by an exponential decay (shock); and a more uniform, gradual rise to peak pressure followed by a near-symmetrical decay (bubble). Shock-type loading resembles an air shock with a discrete, low-magnitude particle barrage overlain, and is caused by premature rupture of the soil bubble and venting of the previously confined detonation products. Bubble-type loading resembles the loading from impact of a pressurized fluid droplet, and is as a result of the soil bubble remaining intact prior to, and for some time during, impingement on the target surface.
— Decreasing SOD focuses the loading more centrally and increases the magnitude of pressure and specific impulse, whereas increasing the SOD results in a more spatially uniform loading distribution, with lower relative values in the centre and larger relative values at the edge of the instrumented region.
— The mechanism of load transfer in saturated soils has been shown to be similar to that produced by an explosive submerged in water. Uniformly graded soils (e.g. LB) have also been shown to produce a repeatable loading distribution, whereas well-graded soils (e.g. Stanag, as currently specified in AEP-55 [29]) exhibit greater variability and feature larger, discrete particle strikes which serve to considerably increase localized specific impulse.
— Bulk density appears dominant in determining the temporal characteristics of the loading, such as arrival time and duration, for saturated soils.

The results herein demonstrate the complexity of the loading generated from a shallow-buried explosive, and highlight critical mechanisms which drive both the form and magnitude of the imparted load. Detailed knowledge of such loading is critical in enabling engineers to design efficient and effective protective structures, and also provides a wealth of information for rigorous validation of existing and newly developed numerical modelling approaches [24,45,46]. Findings relating loading variability to both moisture content and PSD have implications for academic research and future revisions of testing standards, e.g. [29,30], and provide an important benchmark on the achievable level of experimental control and test-to-test variability.

## Footnotes

1 This height was adjusted for Series H and I, as detailed in table 1.

2 In this paper, ‘STANAG’ refers to the NATO standardization agreement, whereas ‘Stanag’ refers to the well-graded sandy gravel used in the current testing.

3 Previous unpublished work has shown that the steel container offers no confinement to the early-stage expansion of the detonation products. In this study, the container has been retained to offer a means to support the charge beneath the target plate; this was achieved by placing the charge on a small timber prop within the container.

4 Analogous to arrival time.

## Data accessibility

Experimental data have been provided in a comprehensive format as part of the electronic supplementary material available alongside this paper with a view to facilitating future studies into predicting and protecting against the effects of high explosives buried in soil.
